# Cremation

**Published:** 1888-10

**Authors:** 


### CREMATION.

An English clergyman, the Rev. C. J. Street, M. A., says the *Sanitary News*, has been reading a paper on cremation at Croydon, and he not only approved, but championed, what, at one time, was supposed to be an anti-christian custom. Death, he said, was the source of life, and the dissolution of the one generation was necessary for the health of the succeeding generation. All dead bodies resolved themselves into their constituent elements; but they were doing their best to thwart and hinder this process by the present system of burial, more especially if they yielded to the folly of leaden or oak coffins. In cremation the work was done in a single hour, and without the dangers which accompanied the present system. Recent science showed that the most devastating diseases were due to living organisms which fed upon the victim. These disease germs continued to live in the air long after the body had ceased to exist.

When graves were opened exhalations were allowed to get out. Nay, it was known that paupers' graves had been opened and the bones taken out and burned, the graves being again used for a similar purpose as before. It was related that soil from a graveyard sprinkled on a garden thirty years after it became infected had been the means of creating disease. When these facts were borne in mind, and it was also remembered that in England and Wales there were 11,000 cemeteries, they need not wonder at the continual scourges of the epidemic diseases which beset our country. In 1874 it transpired that the entire drainage of a cemetery discharged itself into the river Wandel, from which some of the people of Tooting were in the habit of taking their water. In 1851 the average cost of cemetery land per acre was shown to be £123. It was said this was no waste of land, because cemeteries made public parks, but he did not share in the taste which delighted in taking a "constitutional" in the neighborhood of tombstones. He preferred to walk through the crowded streets. Some people opposed cremation because they said it would prevent the Lord raising them at the Last Day. This was, of course, based upon ignorance. Surely the day was past for men seriously to maintain that the actual body that died would be raised particle for particle on the resurrection day! The present Bishop of Manchester held this to be a degrading superstition. Bishop Fraser concurred in that opinion, and it was condemned by all right thinking people. Cremation would prevent two things: The robbery of dead bodies, and the possibility of being buried alive. If, by chance, a live body should be cast into the furnace death would be almost instantaneous, unlike the slow torture if such an event happened by burial. In the discussion which followed the only dissenting voice heard was that of a doctor who thought cremation would shield the secret poisoner.

